# Assessing Interstimulus Interval and Waveform Effects on Vibrotactile Pattern Recognition on the Forearm for Transfemoral Prosthetic Sensory Feedback

**DOI:** 10.3390/s26092664

**Published:** 2026-04-25

**Authors:** Mohammadmahdi Karimi, Kristín Briem, Árni Kristjánsson, Sigurður Brynjólfsson, Runar Unnthorsson

**Affiliations:** 1 School of Engineering and Natural Sciences, University of Iceland, 102 Reykjavík, Iceland; sb@hi.is (S.B.); runson@hi.is (R.U.); 2 School of Health Sciences, University of Iceland, 102 Reykjavík, Iceland; kbriem@hi.is (K.B.); ak@hi.is (Á.K.)

**Keywords:** haptic feedback, sensory substitution, vibrotactile interface, wearable tactile technology

## Abstract

Providing reliable sensory feedback is one of the most challenging aspects of transfemoral prosthetics, motivating the development of intuitive vibrotactile interfaces capable of conveying information about limb position in real-time. The aim of this study was to develop a vibrotactile feedback prototype and examine which interstimulus intervals (ISIs) and vibration waveforms might best enhance recognition of sequential tactile patterns. The results will be used to inform the development of a prototype to be tested on participants with transfemoral amputation where prosthetic feedback is provided. A forearm-mounted six-actuator feedback system, encoding eight lower-limb configurations, was used in two experiments with healthy adults. Experiment 1 assessed recognition accuracy across ISIs from 10 to 110 ms, while Experiment 2 compared sinusoidal and square waveforms under matched conditions. Recognition accuracy was high across all tested conditions, with no significant effects of ISI (*p* = 0.79) or waveform type (*p* = 0.17). These results indicate that participants were able to interpret spatially distributed vibrotactile patterns even under rapid temporal sequencing and with differing signal shapes. The system therefore offers design flexibility for real-time prosthetic feedback, suggesting that fast update rates may be achievable without a statistically detectable reduction in perceptual clarity within the tested conditions. These findings provide practical guidance for developing robust, user-friendly sensory substitution systems intended to increase proprioceptive awareness in transfemoral prosthesis users.

## 1. Introduction

Restoring sensory feedback in lower-limb prostheses is one of the most persistent challenges in prosthetic design [1,2]. While current transfemoral prostheses incorporate sophisticated mechanical and control features that enable near-natural motion [3,4], they still fail to provide the user with proprioceptive information about limb state or movement [5,6]. The absence of such feedback forces users to rely primarily on visual cues to monitor their prosthesis [7], increasing attentional demands and reducing the fluidity and safety of movement [8]. A robust, intuitive feedback system capable of conveying proprioceptive information through another sensory channel could therefore improve stability, safety, and user confidence during gait, and potentially increase prosthesis embodiment [9].

Vibrotactile feedback systems have shown promise for this purpose [9]. By translating prosthetic sensory data into localized vibrations on the skin, they can noninvasively communicate spatial or temporal aspects of limb movement. Previous studies have demonstrated that vibrotactile cues can effectively support gait phase detection [10,11,12,13,14], postural control [15,16,17], and joint position awareness [18,19,20] in both amputee and non-amputee populations. However, the literature also reveals considerable variation in perceptual performance depending on the characteristics of the vibration signal, suggesting that some questions about optimal parameter selection remain [9].

Successful implementation of such feedback systems, however, is contingent upon optimizing the design parameters that govern perceptual clarity and discrimination accuracy. The interaction of these parameters has recently been examined in wearable haptic systems designed for feedback in different applications [21,22]. Nevertheless, relatively few studies have systematically examined how temporal parameters, such as interstimulus intervals (ISIs), and signal characteristics, like waveform shape, jointly influence recognition accuracy in multi-actuator wearable interfaces.

In our previous work, we introduced a forearm-mounted vibrotactile feedback system designed to provide transfemoral prosthesis users with proprioceptive information about lower-limb joint configurations. The system was tested on healthy subjects where assumed (prosthetic) knee and ankle positions were mapped via vibrotactile stimuli to dorsal and volar locations on the forearm [23]. The forearm was selected because it is a relatively passive body region that does not interfere with locomotion or other functional tasks [21]. Moreover, prior studies have demonstrated that the forearm provides relatively good spatial acuity and consistent localization of vibrotactile stimuli [24]. The feasibility of embedding vibrotactile feedback devices within the prosthetic socket for all transfemoral prosthesis users is currently unknown. Socket-integrated solutions may pose practical challenges related to fit, comfort, and long-term usability, and their effectiveness is likely to vary across individuals. In contrast, an externally mounted feedback system, such as a forearm-mounted interface, may offer a more flexible and broadly applicable alternative in cases where socket-based stimulation is impractical or undesirable. For this reason, the present work focuses on a generalizable forearm-mounted vibrotactile system for delivering prosthetic sensory feedback.

The present study aimed to develop a forearm-mounted vibrotactile feedback system and to conduct two controlled laboratory experiments to address the following research questions: (1) What is the shortest tested ISI that allows reliable recognition of sequential vibrotactile stimulation on the forearm? (Experiment 1) and (2) Do square and sinusoidal signals differ in discrimination performance when all other stimulation parameters are held constant (Experiment 2)? The independent variables were ISI in Experiment 1 and waveform type in Experiment 2. The dependent variables were participants’ recognition accuracy for the vibrotactile patterns and their response time. Shorter ISIs were hypothesized to potentially reduce recognition accuracy due to potential temporal overlap between signals, while square waveforms were hypothesized to produce slightly higher accuracy because of their sharper onset profiles. The results will inform and optimize the design of sensory feedback systems intended for prosthetic use and serve as the basis for guidelines regarding timing and signal selection in real-time feedback applications. Such guidelines will help ensure that tactile cues remain perceptually distinct even at high update rates. Our hope is that the study will contribute to the broader goal of developing efficient, intuitive, comfortable, and effective vibrotactile interfaces for proprioceptive feedback and to improve functional outcomes and safety for transfemoral prosthesis users.

## 2. Materials and Methods

### 2.1. Participants

Twelve healthy adults participated in Experiment 1 (10 males and 2 females; mean age = 30.5 years, standard deviation (SD) = 5.7), while a separate cohort of twelve individuals took part in Experiment 2 (9 males and 3 females; mean age = 31.5 years, standard deviation (SD) = 5.3). Six individuals completed both experiments, while six participated only in Experiment 1 and six participated only in Experiment 2. All participants gave written informed consent before the experiments started. Inclusion criteria for participants were healthy adults (18 years or older) who were able to understand the study procedures. Participants were excluded if they reported any neurological disorder, tactile sensory deficit, or uncorrected vision problem that could affect perception of the vibrotactile stimuli or completion of the task. The research protocol was reviewed by the National Bioethics Committee (reference VSN2501016, dated 29 January 2025), which concluded that formal approval was not required. The study was nevertheless conducted in full accordance with institutional ethical standards and the principles of the Declaration of Helsinki.

### 2.2. Apparatus

**Figure 1:**
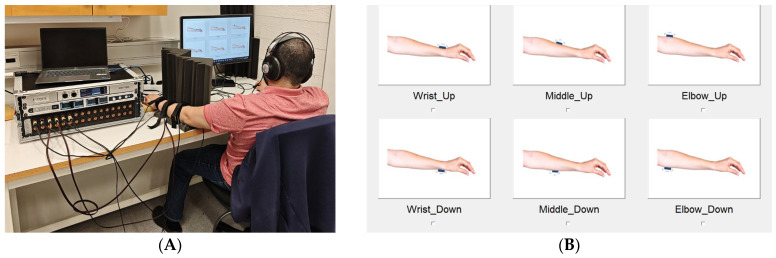
The forearm-mounted vibrotactile system used in the experiments. (**A**) Participant setup showing the placement of the three adjustable bands on the left forearm. (**B**) The graphical user interface used for reporting perceived vibrotactile patterns.

The custom-designed forearm-mounted vibrotactile feedback system consisted of three adjustable bands made from polyester webbing with hook-and-loop fasteners, positioned on the left forearm at three sites: just above the wrist, at the midpoint between wrist and elbow, and just below the elbow (Figure 1). We ensured that the middle band was positioned at the midpoint between the wrist and elbow bands, providing consistent relative spacing and alignment across participants. Each band housed two L5 (Lofelt GmbH, Berlin, Germany) voice-coil actuators positioned on the dorsal (posterior) and volar (anterior) surfaces of the forearm and encased in 3D-printed housings that ensured consistent contact and comfort. Building on earlier prototype development [23], the actuators were mapped to represent different lower-limb joint configurations of an assumed transfemoral prosthesis. A ‘Toe-off’ was signaled by activation of the dorsal actuator on the wrist band, and a ‘heel strike’ corresponded to the volar actuator on the same band. On the middle band, the dorsal actuator indicated ankle dorsiflexion and the volar actuator indicated plantarflexion. At the elbow band, knee extension and flexion were represented by dorsal and volar actuators, respectively. These six actuators were therefore combined to produce eight distinct vibrotactile encoding patterns (Table 1), each corresponding to a unique configuration of lower-limb positions. The signals were generated using custom Python software (version 3.12.3) and delivered via an RME MADIface XT digital audio interface (RME GmbH, Chemnitz, Germany) and Ferrofish A32 converters (Ferrofish GmbH, Linz am Rhein, Germany). The experimental software was run on a Dell Latitude 7440 laptop (13th Gen Intel Core i7-1365U processor, 32 GB RAM, 64-bit Windows 11 Enterprise). The Python software controlled stimulus generation, timing, randomization, and response logging within a unified experimental framework.

Multi-channel amplifiers powered the actuators and no vendor-specific Python Application Programming Interface for the RME MADIface XT was used. In the custom Python implementation, the six stimulation channels were generated as independent audio output channels using NumPy 2.1.1 for waveform synthesis and the Sounddevice 0.4.1 library in Audio Stream Input/Output mode for multichannel streaming. Each channel carried either a 200 Hz sinusoidal waveform or a square waveform of the same fundamental frequency, while inactive channels were set to zero amplitude. The output stream was configured as a single synchronized six-channel audio stream at a sampling rate of 44,100 Hz, so that all channels shared the same hardware clock and remained temporally aligned. Signals were sent from the laptop via USB to the RME MADIface XT, routed digitally through the MADI link to the Ferrofish A32, converted to analog outputs, and then passed to the multi-channel amplifiers that drove the six voice-coil actuators. All channels were assigned to fixed hardware outputs, and gain settings were kept constant across conditions. Amplitude was matched at the digital output level, with no additional per-actuator mechanical calibration applied. In Experiment 1, sequential activation timing was controlled in software by presenting the three active actuators in fixed distal-to-proximal order (wrist, middle forearm, elbow), with 200 ms stimulus duration per actuator and a programmed randomized interstimulus interval of 10–110 ms between activations; in Experiment 2, the same structure was used with a fixed 50 ms interstimulus interval. Because all six outputs were generated within the same audio callback and delivered through one interface stream, synchronization between channels was maintained within each trial. Participants interacted with the system through a graphical user interface (GUI) displayed on a computer screen, which allowed them to record their responses using a standard computer mouse (Figure 1).

### 2.3. Procedure

On arrival, participants were briefed on the aims and procedures of the study and fitted with the three vibrotactile bands. They were seated with their left arm resting comfortably on a cushion (Figure 1A), and the system was adjusted to maintain stable contact between actuators and skin. To minimize extraneous cues, participants wore headphones that delivered continuous white noise, and a visual barrier prevented them from seeing their forearm during stimulation. A familiarization phase preceded the experiments, during which participants were exposed to the actuator layout and patterns until they were able to reliably associate each actuator with its corresponding mapped location. Once familiarization was complete, the main experimental sessions began. In both experiments, vibrotactile stimuli were always presented sequentially in the following order: wrist band, then middle band, followed by the elbow band. This ordering was selected to ensure consistent progression from distal to proximal cues corresponding to the represented ankle and knee states. During each trial, only one actuator per band was active, and participants were required to identify the perceived pattern using the GUI (Figure 1B). They were given five seconds to respond after each sequence, although the system advanced to the next trial immediately if a response was recorded earlier. At the end of the session, participants completed a short questionnaire that captured their subjective impressions of the system, primarily to provide additional context for interpretation of the findings (see Appendix A and Appendix B).

### 2.4. Experimental Parameters

Experiment 1 was designed to investigate the influence of the ISI on recognition accuracy. Stimuli were presented with ISIs randomly ranging between 10 ms and 110 ms in 20 ms increments. Each of the eight encoded patterns was repeated five times at each interval, and both ISI and pattern presentation were randomized to reduce predictability. The signals consisted of sinusoidal waveforms delivered at 200 Hz, chosen on the basis of previous research showing high perceptual sensitivity at this frequency [25]. Amplitude was set to the maximum available level (100%), and each signal lasted 200 ms, consistent with our prior work [23].

Experiment 2 examined the effect of waveform type on perceptual discrimination while holding other parameters constant. The ISI was fixed at 50 ms, and two waveform types, sine and square waveforms, were compared. To counterbalance order effects, half of the participants started with the sine waveforms and the other half began with square waveforms. Each waveform condition involved ten randomized presentations of the eight patterns. All other properties, including mapping, amplitude, frequency, and signal duration, were identical to those used in Experiment 1. In the present study, the square waveform was generated digitally as the sign of a 200 Hz sinusoid, resulting in a symmetric bipolar square wave with a 50% duty cycle. The sinusoidal waveform was generated at the same fundamental frequency. In both cases, amplitude was defined in the software as normalized full-scale output (100% of the selected digital output level), with identical gain settings maintained across conditions. No additional digital filtering, envelope shaping, or waveform-specific post-processing was applied in the software before transmission to the audio interface. As with any physical actuator system, the final mechanical vibration at the skin was influenced by the frequency response of the digital-to-analog converter, amplifier, and voice-coil actuator chain.

### 2.5. Data Collection and Analysis

After each stimulus sequence, participants identified the perceived pattern by clicking with a standard computer mouse on the individual actuator locations in the GUI that they believed had been activated. For each trial, the recorded response pattern was compared with the stimulus pattern that had actually been presented. A response was classified as correct only when all selected actuator locations matched the presented pattern exactly; otherwise, it was classified as incorrect. Recognition accuracy was then calculated as the proportion of correct responses for each participant under each experimental condition. Responses were recorded automatically by the computer system, which logged all relevant parameters, including ISI, waveform type, participant responses, and response time for each trial. Recognition accuracy and response time were separately analyzed using linear mixed-effects models (LMMs) within an analysis of variance (ANOVA) framework in R version 4.5.0 using the lme4 package, with the significance threshold set at α = 0.05. To account for repeated observations from the same participants, individual participants were modeled as random effects. In Experiment 1, ISI was modeled as a fixed effect, and in Experiment 2, waveform type was modeled as a fixed effect. For both experiments, model assumptions were evaluated before inferential testing. Normality of residuals was assessed using the Shapiro–Wilk test, and homoscedasticity was assessed using the Breusch–Pagan test. In both experiments, the assumptions were satisfied. In Experiment 1, the model tested the effect of ISIs, whereas in Experiment 2 the focus was on the effect of waveform type.

Effect sizes and 95% confidence intervals (CIs) are reported alongside *p*-values to clarify the magnitude and precision of observed differences. Although the sample size was moderate, the repeated-measures design with many trials per participant (Experiment 1: 240 trials/participant; Experiment 2: 160 trials/participant) increased the amount of data available for analysis. The number of participants is consistent with previous vibrotactile discrimination studies addressing similar questions. This allows us to evaluate whether any observed differences are not only statistically detectable, but also practically meaningful. Nevertheless, the study may have had limited sensitivity to detect small effects, and the results should therefore be interpreted primarily as evidence that no statistically significant differences were observed under the tested conditions.

## 3. Results

### 3.1. Experiment 1

**Figure 2:**
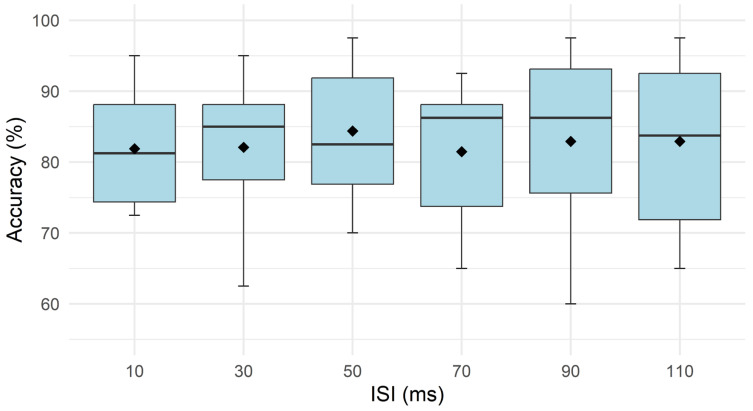
Recognition accuracy across interstimulus intervals (ISIs) of 10–110 ms in Experiment 1. The solid line represents the median accuracy at each interval, while the diamond indicates the mean accuracy.

**Figure 3:**
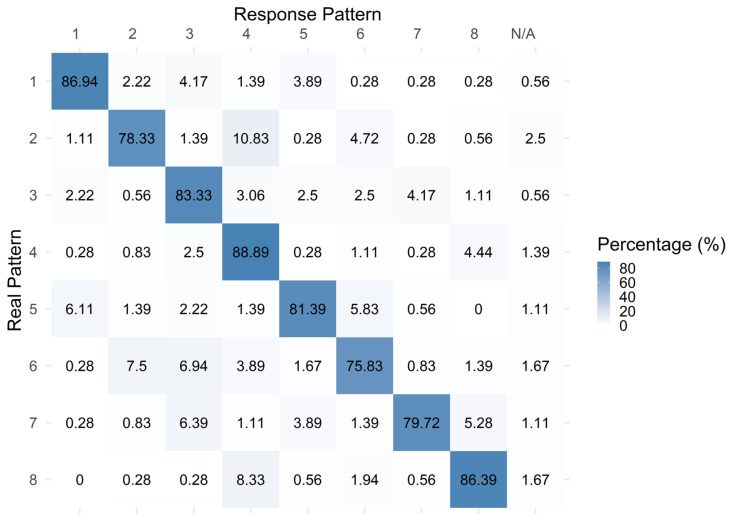
The pattern numbers confusion matrix for Experiment 1 (values are % of responses). Responses that did not match any of the eight valid patterns were grouped as “N/A”.

Recognition accuracy was high across all tested ISIs, with mean values ranging from 81.5% to 84.4% (10 ms: 81.9%, CI [76.8, 87]; 30 ms: 82.1%, CI [75.7, 88.5]; 50 ms: 84.4%, CI [78, 90.8]; 70 ms: 81.5%, CI [74.9, 88.1]; 90 ms: 82.9%, CI [74.5, 91.3]; 110 ms: 82.9%, CI [75.6, 90.2]). No statistically significant effect of ISI was found for accuracy (*p* = 0.79, partial η^2^ ≈ 0.04), indicating that recognition accuracy did not vary significantly across the tested ISIs (Figure 2 and Figure 3).

The confusion matrix (Figure 3) for Experiment 1 showed a clear concentration of responses along the main diagonal, indicating that most patterns were correctly identified. Misclassifications were relatively infrequent and distributed across a limited number of off-diagonal cells, while ‘N/A’ responses were rare, suggesting that participants generally perceived the presented patterns as belonging to one of the eight valid stimulus configurations.

**Figure 4:**
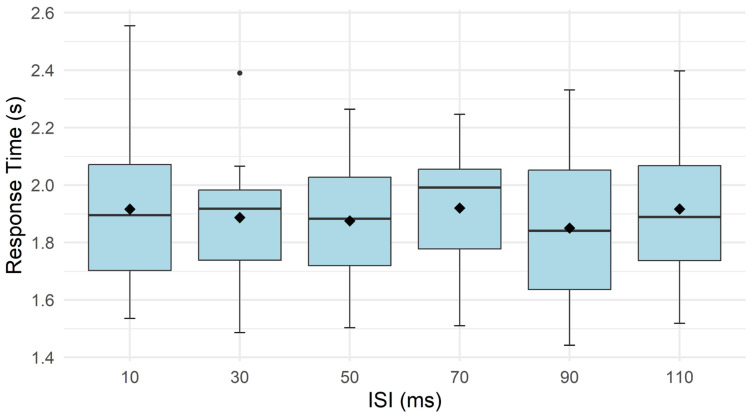
Response time across interstimulus intervals (ISIs) of 10–110 ms in Experiment 1. The solid line represents the median response time at each interval, while the diamond indicates the mean response time, and individual dots denote outliers.

Response time remained relatively stable across all tested ISIs, with mean values ranging from 1.85 s to 1.92 s (10 ms: 1.92 s, CI [1.72, 2.12]; 30 ms: 1.89 s, CI [1.74, 2.04]; 50 ms: 1.88 s, CI [1.73, 2.03]; 70 ms: 1.92 s, CI [1.79, 2.05]; 90 ms: 1.85 s, CI [1.67, 2.03]; 110 ms: 1.92 s, CI [1.77, 2.07]). No statistically significant effect of ISI was found for response time (*p* = 0.49, partial η^2^ ≈ 0.08) (Figure 4).

Questionnaire responses from Experiment 1 indicated that participants generally found vibrotactile patterns easy to distinguish and the device comfortable to wear, while reporting little physical discomfort. Participants also rated the timing of the vibrations as appropriate and indicated that shorter ISIs caused only limited confusion.

### 3.2. Experiment 2

**Figure 5:**
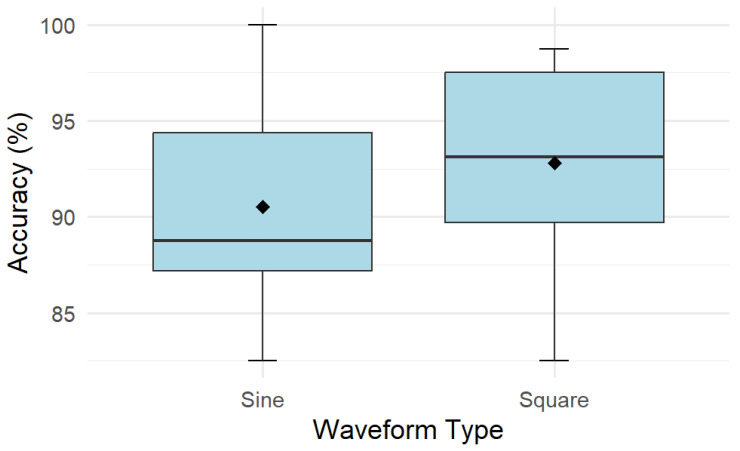
Recognition accuracy for sine and square waveform conditions in Experiment 2. The solid lines indicate median accuracy, and the diamond represents mean accuracy for each waveform type.

**Figure 6:**
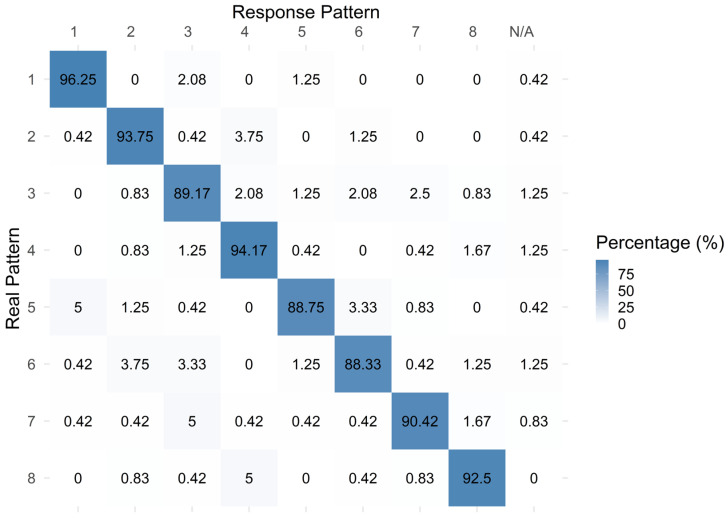
The pattern numbers confusion matrix for Experiment 2 (values are % of responses). Responses that did not match any of the eight valid patterns were grouped as “N/A”.

Recognition accuracy was high for both signal types, with mean values of 90.5% (CI [87, 94]) for sine waveforms and 92.8% (CI [89.5, 96.1]) for square waveforms (Figure 5 and Figure 6). Statistical analysis found no significant difference in accuracy between the two signal types (*p* = 0.17, partial η^2^ ≈ 0.17).

Similarly, the confusion matrix (Figure 6) for Experiment 2 showed a strong diagonal pattern, consistent with the high recognition accuracy observed for both waveform conditions. Off-diagonal responses were limited, indicating only occasional confusion between patterns, and ‘N/A’ responses occurred rarely.

**Figure 7:**
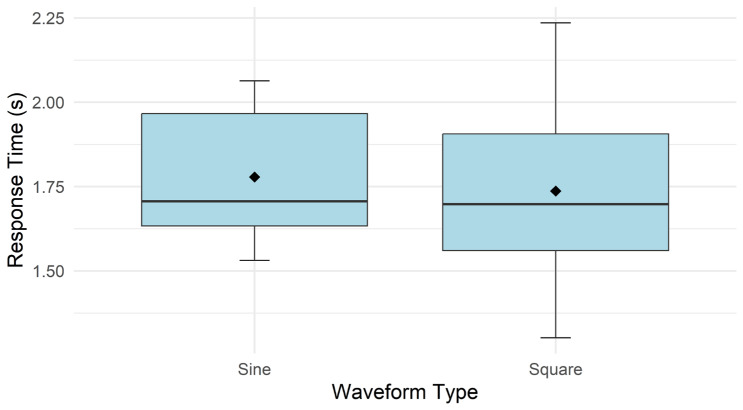
Response time for sine and square waveform conditions in Experiment 2. The solid line represents the median response time at each interval, while the diamond indicates the mean response time.

Response time was stable for both signal types, with mean values of 1.78s (CI [1.65, 1.91]) for sine waveforms and 1.74s (CI [1.56, 1.92]) for square waveforms. Statistical analysis found no significant difference in response time between the two signal types (*p* = 0.34, partial η^2^ ≈ 0.08) (Figure 7).

Questionnaire responses from Experiment 2 indicated that participants reported low levels of physical discomfort and found the vibrotactile patterns easy to distinguish. They perceived only modest differences between sine and square waveforms overall, although square waveforms were preferred more often than sine waveforms.

## 4. Discussion

The present study investigated how ISIs and vibration waveform shapes may influence pattern recognition in a multi-actuator vibrotactile interface intended for transfemoral prosthetic feedback. In contrast with our expectations, the manipulation of neither parameter produced a statistically significant change in recognition accuracy. Both experiments yielded high overall performance levels of study participants, indicating that the vibrotactile system can convey complex spatial patterns reliably even under rapid temporal sequencing and with varying signal shapes.

In Experiment 1, accuracy remained stable between ISIs of 10 ms and 110 ms, suggesting that participants in this study were able to process sequential tactile inputs within this range under the tested laboratory conditions. This finding implies that close temporal spacing between adjacent stimuli did not result in a statistically detectable reduction in recognition performance, possibly because each vibration was spatially distinct and temporally well defined. Our result is consistent with previous research, where even larger ISIs have been tested. Shimizu et al. [26] contrasted ISIs of 120 ms and 300 ms in a tactile point-pressure brain computer interface and found reliable performance with no significant accuracy differences between the two conditions. Yeganeh et al. [27] presented sequential vibrotactile patterns using ISIs from 10 ms to 2500 ms and found optimal performance to be within the range of approximately 50–700 ms for short patterns and 100–500 ms for long patterns. Complementing these findings, Boldt et al. [28] studied sequential electrotactile stimuli with ISIs between 20 ms and 200 ms and found that spatial discrimination significantly improved when the ISIs reached 120 ms or longer, reflecting temporal thresholds in tactile processing. Participants’ subjective feedback supported our findings since they mostly described the signal timing as clear and reported no noticeable confusion even at shorter intervals (see Appendix A), indicating that rapid presentations were still clearly distinguishable. For real-time prosthetic use, this temporal resilience provides design flexibility, and updates may be achievable at relatively high frequencies without a statistically significant reduction in perceptual clarity within the tested conditions. Furthermore, the lack of a decline in accuracy at shorter ISIs suggests that attentional or cognitive load did not substantially increase with faster presentation, an important consideration for long-term usability.

In Experiment 2, although square waveforms showed slightly higher mean accuracy, the difference was small and not statistically significant, suggesting at most a modest advantage in detectability. This outcome is consistent with prior findings in electrovibration research. Vardar et al. [29] reported that although square waves can be perceived as significantly stronger than sinusoidal signals, particularly at frequencies lower than 60 Hz, the benefit diminishes at higher frequencies where perceptual sensitivity to waveform shape converges. Similarly, Jiao et al. [30] found that square, sinusoidal, triangle, and sawtooth waveforms produced comparable absolute detection thresholds, with no statistically significant differences between them, despite small numerical advantages for square waves. Subjective responses from the post-session questionnaire revealed that several participants perceived square wave signals as more distinct and slightly stronger, which may explain their small performance advantage. Conversely, some judged the sine wave vibrations as smoother and more comfortable over repeated trials (see Appendix B). However, the minimal difference observed here implies that when amplitude and frequency are optimized, both waveform types can support high recognition performance. The slight advantage of square waves may nonetheless justify their use in practical applications, particularly where rapid onset perception is beneficial, such as for signaling discrete gait events. It is also possible that the high amplitude and consistent actuator contact used in this experiment minimized the potential influence of waveform shape. Under lower-intensity conditions or when skin–actuator coupling is less stable, waveform-dependent effects might become more pronounced.

**Figure 8:**
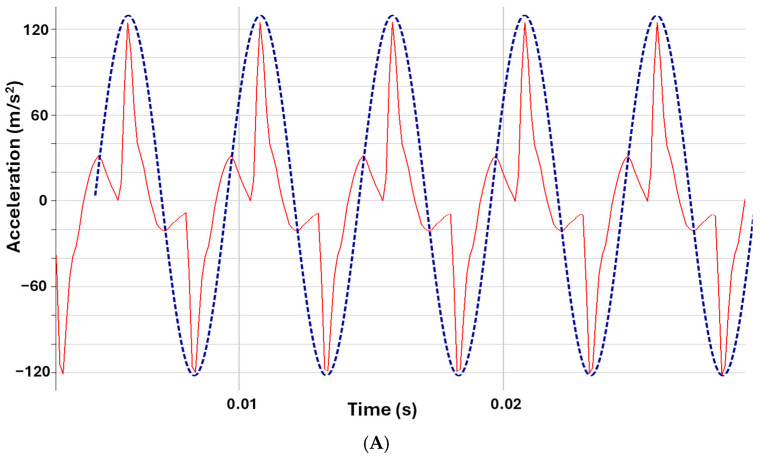

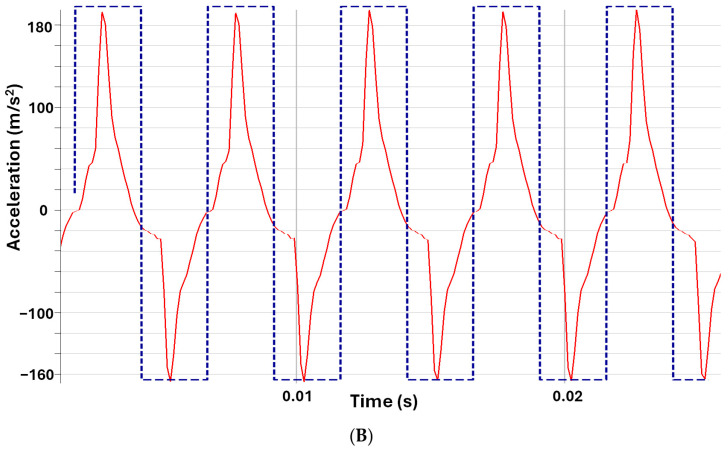
Comparison of commanded and measured vibrotactile signals. (**A**) Sine input waveform (blue dashed line) and corresponding actuator output (red solid line). (**B**) Square input waveform (blue dashed line) and corresponding actuator output (red solid line). Measurements show that while the dominant frequency content is preserved, the mechanical output does not retain the original waveform shape due to the response of the signal chain (audio interface, digital to analog conversion, amplifier, and voice-coil actuator).

It should be noted that independent bench measurements of the actuator output (i.e., not performed on participants), carried out using Brüel & Kjær instrumentation (Nærum, Denmark; LAN-XI data acquisition hardware, Type 3677, with Sensor Type 4520, and BK Connect software 25.1.0.198), show that the commanded waveform shapes (sine and square) are not reproduced exactly at the skin due to the response of the overall signal chain (computer, audio interface, digital-to-analog conversion, amplifier, and voice-coil actuator) (Figure 8). The measurement shown was recorded from one actuator as a representative example of the actuators used in the system, using the same digitally generated 200 Hz sine and square inputs as in the experiments. These traces therefore reflect the transfer characteristics of the complete playback chain rather than the ideal electrical input waveform itself. While the dominant frequency content of the signals is preserved, the resulting mechanical vibrations do not retain the original waveform shapes and include distortions and artifacts. The absence of differences in recognition performance may therefore reflect both perceptual robustness and the characteristics of the physically delivered stimuli. These findings suggest that, within this system, perceptual performance may be primarily influenced by the dominant frequency content of the stimulus rather than the precise temporal waveform. Importantly, this further supports the practical conclusion that precise control of waveform shape is not critical for effective vibrotactile feedback in this system.

The findings of the present study provide direct answers to the two research questions posed in the Introduction. First, the results of Experiment 1 indicate that recognition performance remained high across all tested ISIs, including the shortest tested interval of 10 ms. Under the present conditions, this suggests that temporal spacing within the tested range did not meaningfully limit pattern recognition performance. Second, the results of Experiment 2 indicate that sinusoidal and square waveforms supported similarly high levels of recognition accuracy, with no statistically significant difference between them within the tested conditions.

Together, the findings support the feasibility of implementing compact, temporally dense vibrotactile encoding schemes on a forearm-mounted feedback system for providing transfemoral prosthetic feedback. The high recognition accuracy across all tested conditions indicates that participants were generally able to interpret multi-site vibration patterns even when stimuli are delivered at short intervals and using different waveform types. This flexibility may simplify system design by reducing the need for tight constraints on signal timing or waveform generation, allowing greater focus on optimizing mapping and encoding strategies. From a design perspective, these findings suggest that interstimulus intervals as short as 10 ms may be usable in this system, as no statistically significant reduction in recognition accuracy was observed within the tested range. Additionally, the lack of a strong dependence on waveform type indicates that signal generation can be selected based on practical considerations such as hardware constraints or user comfort. Importantly, the robustness of recognition across both temporal and waveform variations suggests that learning and cognitive adaptation may play a larger role than low-level perceptual limits in determining long-term performance. This is consistent with findings from sensory substitution research, where training and contextual cues often enhance perceptual discrimination beyond initial psychophysical thresholds [21,27,31]. Accordingly, future optimization efforts may be more effectively directed toward encoding strategies, positioning the present results as guidance for prototype development rather than solely evidence of parameter effects.

Despite these encouraging findings, several limitations should be noted when interpreting the results. The study was conducted on able-bodied participants, which may not fully capture the sensory processing characteristics of individuals with transfemoral amputation. Amputation can alter tactile sensitivity, spatial acuity, and cognitive strategies for interpreting feedback, and these effects may further depend on the underlying cause of amputation (e.g., diabetes) [32], which can also influence sensory function in the upper limbs, potentially affecting generalizability. Additionally, the experiments were conducted under controlled laboratory conditions with participants seated and static. As such, some key aspects of real-world prosthetic use were not represented, including dynamic gait, arm movement, divided attention, and potential variations in actuator–skin contact that may arise during daily activities. Real-world prosthetic use involves continuous motion, varying mechanical loads, and complex multisensory integration demands that could influence perception of vibrotactile cues. The present study should therefore be viewed as a controlled validation step, intentionally designed to isolate perceptual effects of temporal and waveform parameters under stable conditions. Such an approach is essential at this stage of device development, as it enables systematic evaluation of fundamental design choices before introducing additional sources of variability.

## 5. Conclusions

These findings suggest that the vibrotactile interface operates within a perceptually robust range, where variations in ISI and signal form did not compromise the clarity or distinctness within the tested conditions. The ability to maintain high accuracy under rapid stimulus presentation suggests that such feedback may be delivered at high update rates without perceptual interference under the tested conditions, supporting the potential for real-time sensory feedback integration in prosthetic systems. Moreover, the comparable results obtained for sine and square waveforms indicate that both can effectively convey spatial and temporal information, allowing flexibility in system design with respect to signal generation and user comfort. Collectively, these results underscore the reliability, efficiency, and perceptual robustness of the developed vibrotactile system and support its potential use for intuitive sensory feedback in transfemoral prosthesis users.

Future research should evaluate the system in people with transfemoral amputation, and over longer periods of use to better understand adaptation, usability, and real-world performance. Further work may also explore alternative actuator placements and implementation approaches to optimize sensory feedback delivery in prosthetic applications.

## Figures and Tables

**Table 1:** The eight encoding patterns as combinations of activated actuator on the forearm.

Pattern	Wrist	Middle Forearm	Elbow
1	Dorsal	Dorsal	Dorsal
2	Dorsal	Dorsal	Volar
3	Dorsal	Volar	Dorsal
4	Dorsal	Volar	Volar
5	Volar	Dorsal	Dorsal
6	Volar	Dorsal	Volar
7	Volar	Volar	Dorsal
8	Volar	Volar	Volar

## Data Availability

The datasets and custom Python scripts used for stimulus generation and data collection are available from the corresponding author upon reasonable request.

## Abbreviations

The following abbreviations are used in this manuscript:

ISI	Interstimulus Interval
SD	Standard Deviation
GUI	Graphical User Interface
ANOVA	Analysis of Variance
LMM	Linear Mixed-effects Model
CI	Confidence Interval

## Appendix A

**Table A1:** Summary statistics for Experiment 1 questionnaire responses (*N* = 12).

Questions	Scale Anchors	Mean	SD
Q1: How easy was it to distinguish between the different vibrotactile patterns presented in this experiment?	1 = Very difficult 5 = Very easy	4.00	0.60
Q2: How comfortable were the vibration actuators when placed on your forearm during the experiment?	1 = Not comfortable at all 5 = Extremely comfortable	4.25	0.96
Q3: Did you experience any physical discomfort from the stimulation?	1 = None 5 = Severe	1.41	0.66
Q4: How appropriate did the time of vibration feel for distinguishing them?	1 = Not appropriate at all 5 = Extremely appropriate	4.08	0.79
Q5: To what extent did shorter delays between vibrations cause confusion or overlap in your perception?	1 = Not at all 5 = Extremely much	2.08	0.90
Q6: How confident were you in recognizing the order of vibrations when the delay was long?	1 = Not confident at all 5 = Completely confident	3.75	0.62

## Appendix B

**Table A2:** Summary statistics for Experiment 2 questionnaire responses (*N* = 12).

Questions	Scale Anchors	Mean	SD
Q1: Did you experience any physical discomfort from the stimulation?	1 = None 5 = Severe	1.08	0.28
Q2: How easy was it to distinguish between the different vibrotactile patterns presented in this experiment?	1 = Very difficult 5 = Very easy	4.25	0.62
Q3: How different did types of wave vibrations feel from each other?	1 = Not different 5 = Extremely different	2.75	1.13
Q4: Did your ability to recognize the patterns improve over time?	1 = Not at all 5 = Improved a lot	2.83	1.69
Q5: Did you feel fatigued or lose focus during the experiment or over time?	1 = Not at all 5 = Extremely much	2.33	1.15

**Table A3:** Preferred vibration signal type for recognition.

Response Option	Percentage (%)
Sine	16.7
Square	50
No difference	33.3

## References

[1] Kulkarni P.G., Paudel N., Magar S., Santilli M.F., Kashyap S., Baranwal A.K., Zamboni P., Vasavada P., Katiyar A., Singh A.V. (2023). Overcoming Challenges and Innovations in Orthopedic Prosthesis Design: An Interdisciplinary Perspective. Biomed. Mater. Devices.

[2] Mariscal D.M., Driscoll B., Huang H., Fisher L.E. (2025). Somatosensory restoration and neural control strategies in lower-limb prostheses. NPJ Biomed. Innov..

[3] Safari R. (2020). Lower limb prosthetic interfaces: Clinical and technological advancement and potential future direction. Prosthet. Orthot. Int..

[4] Gehlhar R., Tucker M., Young A.J., Ames A.D. (2023). A review of current state-of-the-art control methods for lower-limb powered prostheses. Annu. Rev. Control.

[5] Fagioli I., Mazzarini A., Livolsi C., Gruppioni E., Vitiello N., Crea S., Trigili E. (2024). Advancements and Challenges in the Development of Robotic Lower Limb Prostheses: A Systematic Review. IEEE Trans. Med. Robot. Bio..

[6] Domínguez-Ruiz A., López-Caudana E.O., Lugo-González E., Espinosa-García F.J., Ambrocio-Delgado R., García U.D., López-Gutiérrez R., Alfaro-Ponce M., Ponce P. (2023). Low limb prostheses and complex human prosthetic interaction: A systematic literature review. Front. Robot. AI.

[7] Li M., Zhong B., Liu Z., Lee I.C., Fylstra B.L., Lobaton E., Huang H.H. (2019). Gaze Fixation Comparisons Between Amputees and Able-bodied Individuals in Approaching Stairs and Level-ground Transitions: A Pilot Study. Annu. Int. Conf. IEEE Eng. Med. Biol. Soc..

[8] Manz S., Valette R., Damonte F., Gaudio L.A., Gonzalez-Vargas J., Sartori M., Dosen S., Rietman J. (2022). A review of user needs to drive the development of lower limb prostheses. J. NeuroEng. Rehabil..

[9] Karimi M., Yeganeh N., Makarov I., Sverrisson A.Ö., Gunnarsson K.F., Briem K., Brynjólfsson S., Kristjánsson Á., Unnthorsson R. (2025). Haptic Feedback Systems for Lower-Limb Prosthetic Applications: A Review of System Design, User Experience, and Clinical Insights. Bioengineering.

[10] Plauché A., Villarreal D., Gregg R.D. (2016). A Haptic Feedback System for Phase-Based Sensory Restoration in Above-Knee Prosthetic Leg Users. IEEE Trans. Haptics.

[11] Crea S., Edin B.B., Knaepen K., Meeusen R., Vitiello N. (2017). Time-Discrete Vibrotactile Feedback Contributes to Improved Gait Symmetry in Patients with Lower Limb Amputations: Case Series. Phys. Ther..

[12] Martini E., Cesini I., D’Abbraccio J., Arnetoli G., Doronzio S., Giffone A., Meoni B., Oddo C.M., Vitiello N., Crea S. (2021). Increased Symmetry of Lower-Limb Amputees Walking with Concurrent Bilateral Vibrotactile Feedback. IEEE Trans. Neural. Syst. Rehabil. Eng..

[13] Kalff M.N., Hoursch V., Jopp L., Witowski V., Wilke M., Gardetto A., Eberlin K.R., Sehmisch S., Ernst J. (2024). Impact of Gait-Synchronized Vibrotactile Sensory Feedback on Gait in Lower Limb Amputees. Appl. Sci..

[14] Bowman T., Pergolini A., Carrozza M.C., Lencioni T., Marzegan A., Meloni M., Vitiello N., Crea S., Cattaneo D. (2024). Wearable biofeedback device to assess gait features and improve gait pattern in people with parkinson’s disease: A case series. J. NeuroEng. Rehabil..

[15] Chen L.J., Feng Y.G., Chen B.J., Wang Q.N., Wei K.L. (2021). Improving postural stability among people with lower-limb amputations by tactile sensory substitution. J. Neuroeng. Rehabil..

[16] Ballardini G., Florio V., Canessa A., Carlini G., Morasso P., Casadio M. (2020). Vibrotactile Feedback for Improving Standing Balance. Front. Bioeng. Biotech..

[17] Allum J.H.J., Rust H.M., Lutz N., Schouenborg C., Fischer-Barnicol B., Haller V., Derfuss T., Kuhle J., Yaldizli Ö. (2021). Characteristics of improvements in balance control using vibro-tactile biofeedback of trunk sway for multiple sclerosis patients. J. Neurol. Sci..

[18] Lauretti C., Pinzari G., Ciancio A.L., Davalli A., Sacchetti R., Sterzi S., Guglielmelli E., Zollo L. A vibrotactile stimulation system for improving postural control and knee joint proprioception in lower-limb amputees. Proceedings of the 26th IEEE International Symposium on Robot and Human Interactive Communication (RO-MAN).

[19] Chen B., Feng Y., Wang Q. (2016). Combining Vibrotactile Feedback with Volitional Myoelectric Control for Robotic Transtibial Prostheses. Front. Neurorobot..

[20] Maldonado-Contreras J., Marayong P., Khoo I.-H., Rivera R., Ruhe B., Wu W. Proprioceptive improvements of lower-limb amputees under training with a vibrotactile device—A pilot study. Proceedings of the 2017 IEEE Healthcare Innovations and Point of Care Technologies (HI-POCT).

[21] Kristjánsson Á., Moldoveanu A., Jóhannesson Ó.I., Balan O., Spagnol S., Valgeirsdóttir V.V., Unnthorsson R. (2016). Designing sensory-substitution devices: Principles, pitfalls and potential 1. Restor. Neurol. Neurosci..

[22] Fleck J.J., Zook Z.A., Clark J.P., Preston D.J., Lipomi D.J., Pacchierotti C., O’Malley M.K. (2025). Wearable multi-sensory haptic devices. Nat. Rev. Bioeng..

[23] Karimi M., Brynjólfsson S., Briem K., Kristjánsson Á., Unnthorsson R. (2026). Perceptual Design and Evaluation of a Forearm-Based Vibrotactile Interface for Transfemoral Prosthetic Feedback. Biomimetics.

[24] Elsayed H., Weigel M., Müller F., Schmitz M., Marky K., Günther S., Riemann J., Mühlhäuser M. (2020). VibroMap: Understanding the Spacing of Vibrotactile Actuators across the Body. Proc. ACM Interact. Mob. Wearable. Ubiquitous. Technol..

[25] Ævarsson E.A., Ásgeirsdóttir T., Pind F., Kristjánsson Á., Unnthorsson R. (2022). Vibrotactile Threshold Measurements at the Wrist Using Parallel Vibration Actuators. ACM Trans. Appl. Percept..

[26] Shimizu K., Makino S., Rutkowski T.M. (2015). Inter-stimulus interval study for the tactile point-pressure brain-computer interface. Annu. Int. Conf. IEEE Eng. Med. Biol. Soc..

[27] Yeganeh N., Makarov I., KristjÁNsson Á., Unnthorsson R. (2025). Vibrotactile pattern recognition:Influence of interstimulus intervals. Virtual Real. Intell. Hardw..

[28] Boldt R., Gogulski J., Gúzman-Lopéz J., Carlson S., Pertovaara A. (2014). Two-point tactile discrimination ability is influenced by temporal features of stimulation. Exp. Brain. Res..

[29] Vardar Y., Güçlü B., Basdogan C. (2017). Effect of Waveform on Tactile Perception by Electrovibration Displayed on Touch Screens. IEEE Trans. Haptics.

[30] Jiao J., Wang D.X., Zhang Y.R., Cao D.K., Visell Y., Guo X.W., Sun X.Y. (2019). Detection and Discrimination Thresholds for Haptic Gratings on Electrostatic Tactile Displays. IEEE Trans. Haptics.

[31] Erwin A., Sup F.C. (2015). A Haptic Feedback Scheme to Accurately Position a Virtual Wrist Prosthesis Using a Three-Node Tactor Array. PLoS ONE.

[32] Templeton C.A., Strzalkowski N.D.J., Galvin P., Bent L.R. (2018). Cutaneous sensitivity in unilateral trans-tibial amputees. PLoS ONE.

